# Distinct Murine Pancreatic Transcriptomic Signatures during Chronic Pancreatitis Recovery

**DOI:** 10.1155/2021/5595464

**Published:** 2021-05-15

**Authors:** Yinjie Zhang, Baibing Yang, Joy M. Davis, Madeline M. Drake, Mamoun Younes, Qiang Shen, Zhongming Zhao, Yanna Cao, Tien C. Ko

**Affiliations:** ^1^Department of Surgery, The University of Texas Health Science Center at Houston, Houston, TX 77030, USA; ^2^Department of Pathology & Laboratory Medicine, The University of Texas Health Science Center at Houston, Houston, TX 77030, USA; ^3^Department of Pathology, George Washington University School of Medicine and Health Sciences, Washington, DC 20037, USA; ^4^Department of Genetics, Louisiana State University Health Sciences Center, New Orleans, LA 70112, USA; ^5^Center for Precision Health, School of Biomedical Informatics, The University of Texas Health Science Center at Houston, Houston, TX 77030, USA; ^6^Human Genetics Center, School of Public Health, The University of Texas Health Science Center at Houston, Houston, TX 77030, USA

## Abstract

We have previously demonstrated that the pancreas can recover from chronic pancreatitis (CP) lesions in the cerulein-induced mouse model. To explore how pancreatic recovery is achieved at the molecular level, we used RNA-sequencing (seq) and profiled transcriptomes during CP transition to recovery. CP was induced by intraperitoneally injecting cerulein in C57BL/6 mice. Time-matched controls (CON) were given normal saline. Pancreata were harvested from mice 4 days after the final injections (designated as CP and CON) or 4 weeks after the final injections (designated as CP recovery (CPR) and control recovery (CONR)). Pancreatic RNAs were extracted for RNA-seq and quantitative (q) PCR validation. Using RNA-seq, we identified a total of 3,600 differentially expressed genes (DEGs) in CP versus CON and 166 DEGs in CPR versus CONR. There are 132 DEGs overlapped between CP and CPR and 34 DEGs unique to CPR. A number of selected pancreatic fibrosis-relevant DEGs were validated by qPCR. The top 20 gene sets enriched from DEGs shared between CP and CPR are relevant to extracellular matrix and cancer biology, whereas the top 10 gene sets enriched from DEGs specific to CPR are pertinent to DNA methylation and specific signaling pathways. In conclusion, we identified a distinct set of DEGs in association with extracellular matrix and cancer cell activities to contrast CP and CPR. Once during ongoing CP recovery, DEGs relevant to DNA methylation and specific signaling pathways were induced to express. The DEGs shared between CP and CPR and the DEGs specific to CPR may serve as the unique transcriptomic signatures and biomarkers for determining CP recovery and monitoring potential therapeutic responses at the molecular level to reflect pancreatic histological resolution.

## 1. Introduction

Chronic pancreatitis (CP) is a devastating disease characterized by inflammation, fibrosis, and consequent loss of exocrine and endocrine pancreatic function. The most common etiology is alcohol consumption, known to increase the risk of CP in a dose-dependent manner and shown to be related to over 50% of CP cases [1–5]. CP imposes large burdens on patients and healthcare systems, as the economic burden of CP in the US is estimated to be over $150 million annually [6]. Further, patients with CP are at a higher risk of developing type 3c diabetes and pancreatic cancer [7, 8]. Although initially recognized as a clinical disease entity in 1946 [9], little to no therapeutic advancement has been made over the past six decades, rendering the treatment primarily supportive.

Fundamental to the lack of therapeutic advancement, the pathophysiologic mechanisms underlying pancreatic injury and recovery remain largely undetermined. Animal models continue to serve as a backbone of pancreatic research, as obtaining human pancreatic tissue throughout the disease course is not practicable. Our group has previously demonstrated the pancreas to be capable of recovery from CP lesions in one of the most widely studied animal models, the cerulein-induced mouse model [10]. How pancreatic recovery is achieved, specifically at the molecular level, remains of great interest, as identifying altered specific genes and pathways would increase understanding of the mechanisms and provide a direction for possible therapeutic advancement.

RNA-sequencing (RNA-seq) has been the main approach for studying genome-wide gene expression change and corresponding cellular or phenotypic outcome [11, 12]. Advanced analysis of the transcriptomes derived through RNA-seq provides both the global view and quantitative change of genes in disease versus normal organs. The differentially expressed genes (DEGs) through RNA-seq data analysis reveal exclusive cellular processes during disease progression to further our knowledge of the diseases, with the potential of identifying targetable pathways for therapeutic development [13–15].

We hypothesized that during CP progression and its transition to recovery, pancreatic transcriptomes will undergo an explicit transition pattern to reflect CP recovery, and the key transcriptomic signatures can be used as potential biomarkers for predicting and monitoring such transition and recovery to guide clinical decision-making. In this study, we used RNA-seq to examine the transcriptional changes occurring within the pancreas during the distinct phases of CP injury and the recovery and identified representative sets of up- and downregulated DEGs, which may contribute to CP recovery. These findings provide novel insights into identifying the involved signaling pathways in CP recovery, with potential for developing future interventional therapies leading to improved clinical treatments for CP.

## 2. Materials and Methods

### 2.1. Materials

Cerulein, the decapeptide analog of the potent pancreatic secretagogue cholecystokinin, was purchased from Bachem Americas, Inc. (Torrance, CA).

### 2.2. Animals and *In Vivo* CP and the Recovery Model

All animal experiments were performed in compliance with the approved IACUC protocol from the Animal Welfare Committee of the University of Texas Health Science Center at Houston. Adult male and female C57BL/6 mice were purchased from the Jackson Laboratory (Bay Harbor, ME) and housed in a 23°C ambient temperature, 12 : 12 light-dark cycle facility. Mice were fed standard laboratory chow and given water *ad libitum* and randomly assigned to control or experimental groups.

CP was induced by intraperitoneal injections of cerulein (50 *μ*g/kg, 5 hourly injections/day, 3 days/week, for 4 weeks). Age-matched control mice were given normal saline injections. Pancreata were harvested 4 days after the final injections (CP and control (CON) groups, *n* = 12 mice (half male and half female)/group, total 24 mice for this experiment) and 4 weeks after the final injections (CP recovery (CPR) and control recovery (CONR) groups, *n* = 8 mice (half male and half female)/group, total 16 mice for this experiment). The pancreas samples were fixed in 10% formalin for morphological studies as we previously described [10], frozen in liquid nitrogen, and stored at -80°C for RNA extraction.

### 2.3. RNA Extraction

Total RNAs were extracted from pancreatic tissue samples using RNeasy Plus Universal Mini Kit following the manufacturer's instruction (QIAGEN Inc., Germantown, MD).

### 2.4. Library Construction and RNA-Sequencing

Total RNAs from the female mouse pancreas were designated as CON, CP, CONR, and CPR groups (*n* = 4 mice/group) and used for library preparation and sequencing, which was performed by Novogene Corporation Inc. (Sacramento, CA). Briefly, quality control of total RNA sample was checked, with the RNA integrity number (RIN) ≥ 6.0, OD260/280 = 1.8–2.2, and OD260/230 ≥ 1.8 in all RNA samples. 1 *μ*g of RNA was used for cDNA library construction at Novogene using an NEBNext® Ultra 2 RNA Library Prep Kit for Illumina® (cat NEB #E7775, New England Biolabs, Ipswich, MA) according to the manufacturer's protocol. Briefly, mRNA was enriched using oligo(dT) beads followed by two rounds of purification and fragmented randomly by adding fragmentation buffer. The first-strand cDNA was synthesized using random hexamer primer, after which a custom second-strand synthesis buffer (Illumina), dNTPs, RNase H, and DNA polymerase I were added to generate the second strand (ds cDNA). After a series of terminal repair, polyadenylation, and sequencing adaptor ligation, the double-stranded cDNA library was completed following size selection and PCR enrichment. The resulting 250-350 bp insert libraries were quantified using a Qubit 2.0 fluorometer (Thermo Fisher Scientific, Waltham, MA) and qPCR. Size distribution was analyzed using an Agilent 2100 Bioanalyzer (Agilent Technologies, Santa Clara, CA, USA). Qualified libraries were sequenced on an Illumina NovaSeq 6000 Platform (Illumina, San Diego, CA, USA) using a paired-end 150 run (2 × 150 bases). Approximately 40 million reads were generated from each library.

### 2.5. Processing of the RNA-seq Data and Analysis of Differentially Expressed Genes (DEGs) and Gene Set Enrichment Analysis (GSEA)

We processed the RNA-seq data using the BETSY system [16]. We began by checking quality with FastQC and trimming adapter and low-quality sequence using Trimmomatic [17]. We then aligned the processed short reads to the hg19 reference genome using the STAR aligner [18] and estimated gene expression measured by transcripts per million (TPM) using RSEM [19] and the counts using HTSeq-count [20]. We verified the quality of the data and alignment using Picard [21]. The read counts were then used for calling differential expression by DESeq2 [22], and the differentially expressed genes (DEGs) were determined by the Log2(fold change) > 1 and the false discovery rate (FDR) < 0.05. For gene set enrichment analysis (GSEA), we identified enriched pathways on the significant gene sets by applying the GATHER algorithm [23] implemented in BETSY using pathway annotations from the MSigDB [24].

### 2.6. Quantitative Real-Time PCR (qPCR)

Total RNA samples from both male and female mice were reversely transcribed to cDNA using the SuperScript™ IV First-Strand Synthesis System (Invitrogen, Carlsbad, CA). The qPCR was performed using the TaqMan gene expression master mix and specific gene probe sets as previously described [25]. The probe sets of mouse *transforming growth factor- (Tgf-) beta1* (or *β1*) (Mm01178820_m1), *collagen (Col) 1a2* (Mm00801666_g1), *fibronectin* (*Fn1*) (Mm01256744_m1), *inhibitor of differentiation* (*Id*) *3* (Mm00492575_m1), *growth differentiation factor* (*Gdf*) *10* (Mm01220860_m1), *fibroblast growth factor* (*Fgf*) *21* (Mm00840165_g1), *Hamp2* (Mm07306654_gH), and *18*s (Hs99999901_s1) (Life Technology Co., Grand Island, NY) were used in the study. The *Δ*cycle threshold (Ct) value was acquired by (Ct (gene of interest) − Ct (housekeeping gene, 18s rRNA)). The 2^-(*Δ*Ct)^ value was presented, and the group means of the 2^-(*Δ*Ct)^ value were used to compare among four groups by ANOVA and to compare between male and female of respective groups by *t*-test, using GraphPad 8.0 (GraphPad Software, La Jolla, California). *p* < 0.05 was considered statistically significant.

## 3. Results

### 3.1. CP Injury and Recovery Phases Were Established from a Representative Cerulein-Induced CP Murine Model

**Figure 1:**
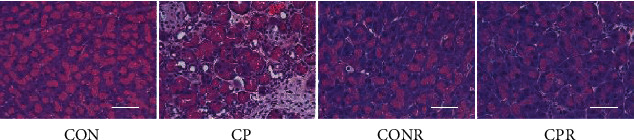
Cerulein-induced CP and the recovery mouse model. Adult C57BL/6 mice, both female and male, received cerulein injections (50 *μ*g/kg, ip, 5 hourly injections/day, 3 days/week) for 4 weeks for CP injury, followed by cessation of cerulein injections for 4 weeks for recovery period (CPR). Time-matched control mice received normal saline injections (CON and CONR). Pancreatic tissue sections were prepared for H&E staining and scored for CP injury as described [10]. Representative images of female mice of CON, CP, CONR, and CPR are shown. Scale bar = 50 *μ*m.

Chronic pancreatitis was induced in adult male and female mice by cerulein (50 *μ*g/kg, intraperitoneal (ip), 5 hourly injections/day, 3 days/week), and the pancreata were harvested 4 days (designated as injury group CP) or 4 weeks (designated as recovery group CPR) after the final injections as previously described and quantified on several major functional analyses [10]. The representative images in Figure 1 demonstrate the morphological changes respective to these groups. Overall, compared to the time-matched controls (CON, CONR), the injury groups displayed CP-induced acinar injury and fibrosis. The recovery groups showed a relatively normal acinar structure with over 50% reduced fibrosis compared with the injury groups. No significant differences between males and females were detected by these functional analyses.

### 3.2. Distinct DEGs Were Identified in CP and the Recovery Pancreas

**Figure 2:**
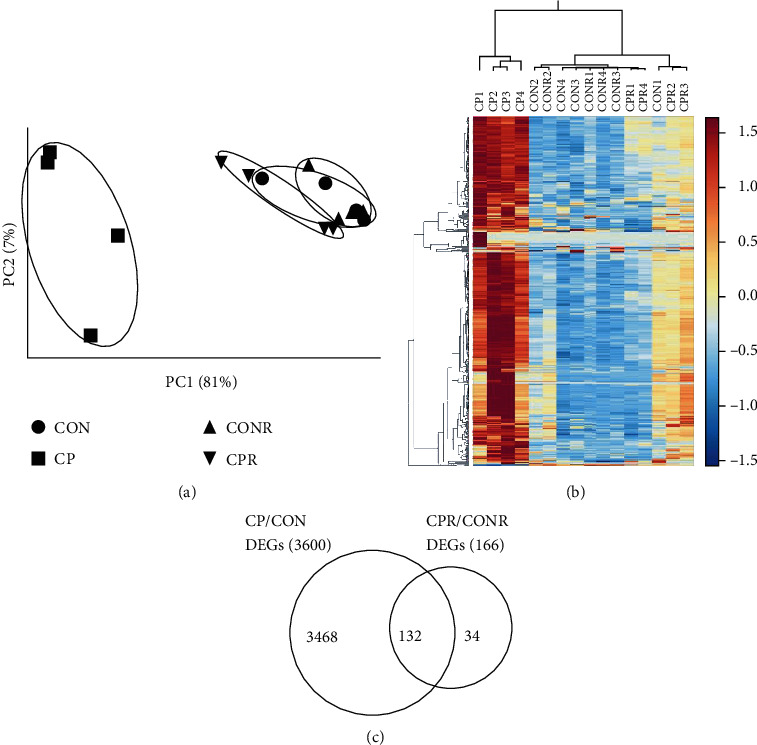
RNA-seq data analysis for the CON, CP, CONR, and CPR groups. Total RNAs were prepared from the mouse pancreata as mentioned in Figure 1, and RNA-seq was performed. (a) Principal component analysis (PCA) illustrates the variance of all genes expressed within each sample and among the groups. (b) The heatmap demonstrates the expression level of the DEGs among samples. Each column is a sample, and each row is a gene. The value is the *z*-score of normalized gene expression counts. For any given gene, the red color represents gene expression that is greater than the overall mean, and the blue color represents gene expression that is less than the overall mean. Hierarchical clustering of genes and samples is represented by the dendrograms on the left and across the top of the heatmap. For the CP and CPR groups, each could cluster as a unique group. (c) The Venn diagram depicts the distribution of the DEGs. The statistical criteria for a gene to be considered differentially expressed were a fold change of Log_2 > 1 and false discovery rate (FDR) < 0.05.

To explore pancreatic recovery after CP induction at the molecular level, RNA-seq was performed to profile pancreatic transcriptomes during CP injury and recovery. Principal component analysis (PCA) was performed on the acquired RNA-seq data for the gross expression patterns among the groups. As shown in Figure 2(a), the CP group displayed as outliers, while the CPR, CON, and CONR groups were clustered together.  Figure 2(b) shows a heatmap of top 500 genes with the highest variability in gene expression for unbiased clustering of samples. Within this heatmap, DEGs in the CP group had higher expression level than other groups, with CON and CONR groups at the lowest and CPR group in the middle. The CP and CPR groups could distinguish themselves from the CON and CONR groups. Compared with the time-matched controls, we identified 3,600 DEGs in CP and 166 in CPR, among which 132 overlapped between CP and CPR, with 3,468 DEGs exclusive to CP and 34 DEGs exclusive to CPR. These results indicate that 4% of DEGs persisted from CP injury to recovery and 34 DEGs newly emerged during CP recovery. A Venn diagram was generated to represent the distribution of the DEGs (Figure 2(c)).

In the 3,468 DEGs exclusive to CP, 3,250 DEGs were upregulated and 218 DEGs were downregulated in the CP group vs. the CON group (Supplementary 1). In the 132 shared DEGs between CP and CPR, 96 DEGs were upregulated and 24 DEGs were downregulated in both CP and CPR; 1 DEG was downregulated in CP while upregulated in CPR; 11 DEGs were upregulated in CP while downregulated in CPR (Table 1). In the 34 DEGs exclusive to CPR, 31 DEGs were upregulated and 3 DEGs were downregulated (Table 2). These results demonstrate the differentially regulated gene expression in the pancreas with CP injury and at the recovery post-CP induction.

### 3.3. Identified RNA-seq Signature Genes Were Validated in Both Female and Male Mice

**Figure 3:**
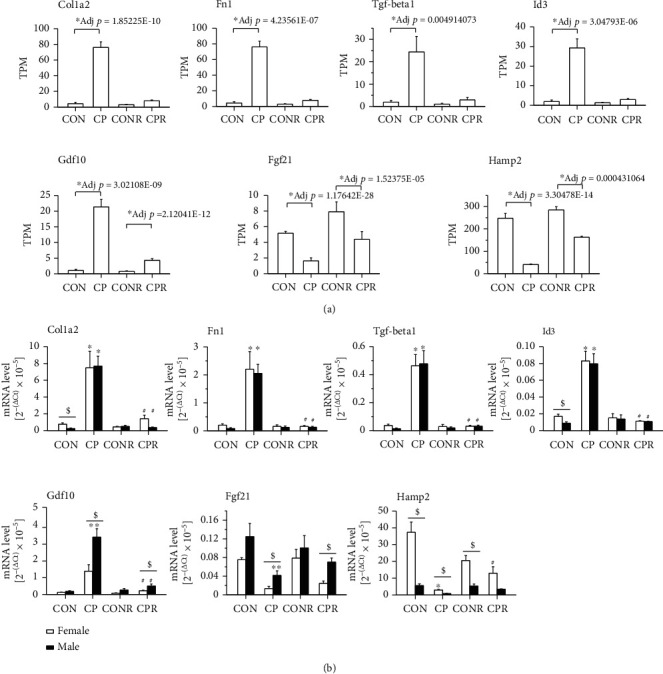
Validation of the selected DEGs from the RNA-seq data by qPCR. (a) The expression levels of the selected DEGs from RNA-seq data. TPM: transcripts per million. RNA-seq data are presented as mean of TPM ± SEM. *n* = 4 mice/group. ^∗^*p* < 0.05 compared between CON and CP, between CONR and CPR. (b) The mRNA level of specific genes by qPCR. The qPCR data are presented as [group means of 2^−(ΔCt)^ ± SEM] × 10^−5^. *n* = 3 − 6 mice/group. ^∗^*p* < 0.05 compared between CON and CP or CONR and CPR. ^#^*p* < 0.05 compared between CP and CPR. ^$^*p* < 0.05 compared between female and male of respective groups.

To validate the RNA-seq data, we utilized RNA samples from both female and male mice for validation and explored sex-dependent gene expression patterns.  Figure 3(a) demonstrates the RNA-seq data of the selected pancreatic fibrosis-relevant genes. Extracellular matrix molecules *Col1a2* [26] and *Fn1* [27], *Tgf-beta1* [28] and one of the downstream molecules *Id3* [29], and *Gdf10* [30] displayed a similar expression pattern, highly upregulated in the CP group and returned to the control levels, except *Gdf10* which was still elevated in the CPR group. However, the RNA-seq data of *Fgf21*, a gene protective in pancreatic fibrosis [31], and *Hamp2*, a gene critical in tissue iron homeostasis [32], exhibited a different expression pattern, dramatically reduced in the CP group and partially recovered in the CPR group. These RNA-seq data were validated by qPCR (Figure 3(b)). For a better understanding of the change in gene expression pattern, we presented the qPCR data as the 2^-(*Δ*Ct)^ value for each gene and individual mouse in the groups (Supplementary 2) with group means in Figure 3(b). We compared CON vs. CP for gene expression in CP injury, CONR vs. CPR for gene expression in CP recovery, and CP vs. CPR for differences between CP recovery and CP injury. We also compared gene expression between female and male mice of respective groups to identify sex-dependent gene expression.

Similar gene expression patterns and levels were observed in female and male mice on *Col1a2*, *Fn1*, *Tgf-beta1*, *Id3*, and *Gdf10*, which were dramatically increased in CP (*p* < 0.05) and declined to control levels or close to control levels in CPR, and consistent with RNA-seq data. Similar gene expression patterns were also observed in female and male mice on *Fgf21* and *Hamp2*, which were dramatically reduced in the CP group (*p* < 0.05) and partially recovered in the CPR group. Furthermore, different expression levels of *Gdf10* and *Fgf21* were identified between female and male mice in the CP and CPR groups and *Hamp2* in the CON, CP, and CONR groups.

In light of clinical implication of our findings derived from mouse models to human diseases, we have searched relevant published articles and databases. We found respective human homologs of 6 out of 7 genes validated in qPCR assays, including ECM genes Col1a2 and Fn1, TGF-beta superfamily and signaling pathway genes Tgf-beta1, Id3, and Gdf10, and Fgf21 (https://www.ncbi.nlm.nih.gov/geoprofiles). Mouse *Hamp2* is unique in mice, and mouse *Hamp1* is the human homolog Hamp. In this study, *Hamp1* level was not significantly altered in CP and CPR respective to controls based on RNA-seq data, thus not validated in qPCR. The expression of these seven genes is variable in normal human pancreas. ECM genes, TGF-beta signaling pathway genes, and Fgf21 remain low expression compared to those in other organs (https://www.ncbi.nlm.nih.gov/geoprofiles), while Gdf10 expression level is the 2^nd^ highest to that of the lungs among over 12 organs tested (https://www.ncbi.nlm.nih.gov/geo/tools/profileGraph.cgi?ID=GDS422:41245_at), and Hamp expression level is the 2^nd^ highest to that of the liver among over 36 organs tested (https://www.ncbi.nlm.nih.gov/geo/tools/profileGraph.cgi?ID=GDS1096:220491_at).

Furthermore, in human CP pancreas, expression levels of ECM and TGF-beta signaling pathway genes are elevated [33, 34]. Taken together, the mouse pancreatic transcriptome in this study largely recapitulates human pancreatic transcriptome. However, the existing differences between mouse and human should be taken into consideration when interpreting data derived from animal studies as well as proceeding to translational studies for human diseases.

### 3.4. Differential Extracellular Matrix (ECM) Remodelling and Cancer Metabolism Gene Sets Were Enriched Using GSEA

**Figure 4:**
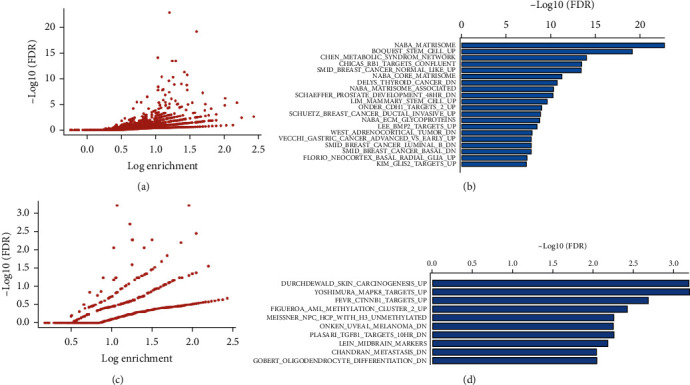
Gene set enrichment analysis of the DEGs identified from CON/CP and CONR/CPR. Distribution of GSEA enrichment FDR versus enrichment *p* value, both with log scale for the (a) 132 common genes between CP and CPR and the (c) 34 unique genes in CPR. (b) The top 20 gene sets enriched in the 132 common genes and (d) the top 10 gene sets enriched in the 34 unique genes, respectively.

To explore the relevant signaling pathways from the DEGs with a focus on pancreatic recovery after CP induction, GSEA was performed for the 132 DEGs that overlapped between CP and CPR, as well as the 34 DEGs exclusive to CPR. The total gene sets generated from the 132 DEGs shared by CP and CPR groups were demonstrated in the plot as shown in Figure 4(a), with the top 20 gene sets shown in Figure 4(b) and the matched DEGs summarized in Table 3. Of note, four gene sets, NABA MATRISOME, NABA_CORE_MATRISOME, NABA_MATRISOME_ASSOCIATED, and NABA_ECM_GLYCOPROTEINS, are relevant to extracellular matrix (ECM) remodelling [35], and nine gene sets, CHICAS_RB1_TARGETS_CONFLUENT [36], SMID_BREAST_CANCER_NORMAL_LIKE_UP [37], DELYS_THYROID_CANCER_DN [38], SCHAEFFER_PROSTATE_DEVELOPMENT_48HR_DN [39], SCHUETZ_BREAST_CANCER_DUCTAL_INVASIVE_UP [40], WEST_ADRENOCORTICAL_TUMOR_DN [41], VECCHI_GASTRIC_CANCER_ADVANCED_VS_EARLY_UP [42], SMID_BREAST_CANCER_LUMINAL_B_DN, and SMID_BREAST_CANCER_BASAL_DN [37], are involved in cancer metabolism. Within the top gene sets, top three regulated DEGs in both CP and CPR are *Cilp* (2.9-fold in CP, 3.2-fold in CPR), *Pgc* (5.3-fold in CP, 2.7-fold in CPR), and *Fetub* (2.9-fold in CP, 2.4-fold in CPR).

Regarding the total gene sets of the 34 DEGs unique to the CPR group, the plot of Figure 4(c) depicts the total gene sets, with the top 10 gene sets further identified in Figure 4(d) and the matched DEGs summarized in Table 4. Among the top 10 gene sets, 2 sets, FIGUEROA_AML_METHYLATION_CLUSTER_2_UP [50]and MEISSNER_NPC_HCP_WITH_H3_UNMETHYLATED [51], are relevant to DNA methylation, while 4 sets, DURCHDEWALD_SKIN_CARCINOGENESIS_UP [52], YOSIMURA_MAPK8_TARGETS_UP [53], FEVR_CTNNB1_TARGETS_UP [54], and ONKEN_UVEAL_MELANOMA_DN [55], are related to cell signaling pathways. Within the top gene sets, top two upregulated DEGs in CPR are *Agr2* (2.9-fold) and *Thbs4* (1.7-fold).

## 4. Discussion

Fibrosis of the pancreas has long been considered an end point of chronic disease. In recent years, studies of chronic pancreatic lesions in experimental animal CP models have revealed regression after withdrawal of insults, as reported by others [60] and our group [10]. These findings provide novel insights and the potential for the therapeutic strategies in improving CP-induced pancreatic fibrosis and function. Based on our prior findings that pancreatic histological lesions in cerulein-induced CP mice recovered after cessation of cerulein [10], in this study, we applied RNA-seq to explore CP recovery at the whole transcriptome level, with the goal of identifying potential therapeutic targets. We identified 132 shared DEGs between the CP and CPR groups, 34 unique DEGs in the CPR group, and generated the respective gene sets based on the relevant functions of these DEGs.

The top 20 gene sets generated from the 132 shared DEGs between CP and CPR are most relevant to ECM remodelling and cancer metabolism (Table 3). The ECM is a fundamental and essential component in the pancreatic stroma that provides architectural and biochemical support to cells. The core structural components of ECM include fibronectins, collagens, laminins, and proteoglycans [61]. Moreover, the ECM serves as a reservoir for growth factors, cytokines, and ECM-remodelling enzymes that collaborate with structural proteins to enhance cell signaling. In addition to its role in normal physiology, alterations of ECM have been associated with various pathologies such as fibrotic diseases, tumor development, and metastasis [62]. ECM is a key regulator of tissue repair and fibrosis development after injury [63]. In the tumor microenvironment, ECM plays an essential role in the development of distant metastasis and specific tumorigenic signaling pathways through modification of cellular adhesion proteins, promotion of angiogenic cytokines, and immune system evasion [64]. The genes involved are differentially expressed in different cancer types and, thus, facilitate metastasis to different organ systems [65].

Furthermore, within the top 20 gene sets generated from the 132 shared DEGs between CP and CPR, we focused on several DEGs that are relevant to ECM remodelling and appear frequently in the top 20 gene sets. Cartilage intermediate layer protein (CILP) is an ECM protein that possesses an antifibrotic activity in ECM remodelling [66]. CILP-1 was reported to inhibit TGF-*β*1-induced Smad3 phosphorylation and the subsequent fibrogenic events during cardiac fibrosis remodelling. It suppressed Smad3 phosphorylation by increasing Akt phosphorylation and therefore promoting the interaction between Akt and Smad3 [66]. In this study, *Cilp* expression was increased 2.9-fold in CP and 3.2-fold in CPR and was involved in 7 out of the top 20 gene sets (Table 3). Gdf10 has been reported to act as a tumor suppressor in mammary epithelial cells that limits proliferation and suppresses epithelial-mesenchymal transition in breast cancer via upregulation of Smad7 [67]. Here, we found that *Gdf10* expression increased in CP and declined to normal level at CP recovery (Figure 3) and was involved in 4 out of the top 20 gene sets (Table 3). These data suggest that *Cilp* and *Gdf10* play important roles in ECM remodelling during the pancreatic repairing process. Fgf21 has been shown to protect the pancreas from inflammation and proteotoxic stress, which was downregulated by oncogenic KRAS in acinar cells to promote pancreatic tumorigenesis in mice [68]. In our study, *Fgf21* was downregulated in CP and recovered partially in CPR (Figure 3) and was involved in 2 of the top 20 gene sets (Table 3), supporting a protective role of Fgf21 in pancreatitis.

For the top 10 gene sets from the 34 DEGs specific to CPR, the top gene sets are related to DNA methylation and signaling pathways (Table 4). DNA methylation is a biochemical process that represses gene transcription. Physiological DNA methylation is crucial for normal development as well as cellular processes such as genomic imprinting, X chromosome lionization, and normal aging. Inflammatory stimuli may lead to aberrant DNA methylation and the altered gene expression [69]. Abnormal DNA methylation has been implicated in pathological processes, including the hypo- and hypermethylation to oncogenes and tumor suppressor genes, respectively [70]. The progressive increase of DNA methylation levels has been associated with the progression of chronic inflammatory diseases developing into cancer [71]. In our study, DNA methylation was involved in 2 out of the top 10 gene sets (Table 4), suggesting that DNA methylation may be critical for disease recovery. Though few genes were reported to be methylated during CP, an increase of genes methylated during the development of pancreatic tumor was widely reported [71]. Relevant to the signaling pathways, the top gene set DURCHDEWALD_SKIN_CARCINOGENESIS_UP [52] involves Podoplanin, a small membrane glycoprotein expressed in cancer-associated fibroblasts, which was reported to contribute to the progression of multiple types of tumors including pancreatic ductal adenocarcinoma [72, 73]. The gene set YOSIMURA_MAPK8_TARGETS_UP [53] contains the c-Jun N-terminal kinase (JNK) pathway, one of the major signaling cassettes of the mitogen-activated protein kinase (MAPK) signaling pathway, which controls a large number of cellular processes, including proliferation, embryonic development, and apoptosis [74]. Inhibition of JNK is reported to be protective in AP [75–77] and CP [78]. The gene set FEVR_CTNNB1_TARGETS_UP [54] includes the Wnt/*β*-catenin signaling pathway, which is critical for the embryonic development of numerous tissues [79] and was reported to be protective in AP [80]. The gene set ONKEN_UVEAL_MELANOMA_DN [55] contains *Id2* and *E-cadherin*. We previously reported that Id2 was a downstream target of TGF-*β* [81], a major profibrogenic factor in the pancreas [82]. TGF-*β* suppressed Id2, and overexpression of Id2 attenuated TGF-*β*-induced apoptosis [81]. E-cadherin is a Ca^2+^-dependent adhesion protein of the classical cadherin family. Upregulation of E-cadherin expression is a protective response and promotes the repair of cell-cell adhesions of pancreatic acinar cells [83].

Furthermore, within the top 10 gene sets generated from the 34 unique DEGs in CPR, we focused on several DEGs that appear frequently in the top 10 gene sets. Thrombospondin-4 (*Thbs4*) is a top DEG upregulated in CPR with 1.7-fold and present in 3 out of the 10 gene sets. Thbs4 is a member of the extracellular calcium-binding protein family, which plays a critical role in cellular adhesion and migration [84]. Anterior gradient 2 (*Agr2*) is another top DEG upregulated in CPR at 2.9-fold. Agr2 has conserved roles in tissue development and regeneration and is positively correlated with human cancers. In the pancreas, Agr2 plays an important role in cancer cell growth and survival, and the expression and secretion of Agr2 is increased during pancreatic tumorigenesis [85, 86]. Our data suggest a promoting role of *Thbs4* and *Arg2* in pancreatic recovery and regeneration.

The regeneration gene family is known to be involved in regeneration of various tissues including pancreas *β* cells. Among this family, regenerating islet-derived 2 (Reg2) is significantly induced in response to diabetes, pancreatitis, high-fat diet, and during pancreatic regeneration [87, 88]. Interestingly, in our study, *Reg2* is the only DEG downregulated in CP but upregulated in CPR (Table 1), suggesting that *Reg2* may suppress inflammation and assist in tissue recovery. The protein Hepcidin-2, which is coded by gene *Hamp2*, is an important regulator of iron entry into cells. Loss of Hepcidin signaling in mice leads to iron overload-induced chronic pancreatitis [89]. Furthermore, bone morphogenic protein (BMP) 2, an antifibrogenic protein in the pancreas [90, 91], and BMP6 have nonredundant roles in Hepcidin regulation of iron [89, 92]. In our study, *Hamp2* was downregulated in CP and CPR (Table 1, Figure 3), suggesting a potential protective role of Hamp2 in CP.

Overall, there are several aspects of novelty in this study. First, comparing the CP injury and recovery phases against their respective time-matched controls allows us to recapitulate a dynamic change of transcriptomes during the disease and the recovery. Second, the unique DEGs identified in the CP recovery imply that the repairing/recovery phase may also be an active process that involves the newly emerging DEGs during the recovery phase. Third, several DEGs identified in the CP recovery are shared with those identified in a cerulein-induced AP recovery mouse model [93], i.e., upregulation of *Cilp* and *Agr2* and downregulation of *Fgf21* and *Hamp2*. These findings intriguingly suggest that there are commonly shared mechanisms that may contribute to AP and CP recovery. Our findings support the speculation that AP and CP, the two most commonly seen pancreatic inflammation lesions, may exist on a spectrum, shedding light on developing disease-specific therapies for AP and CP patients. We acknowledge that there are limitations in this study. For feasibility reasons, only female mouse pancreatic RNA samples were utilized in RNA-seq assays. However, using female mice for RNA-seq experiment is unique because of its contrast to the dominant male animal studies in biomedicine [94]. Furthermore, the RNA-seq data were validated using qPCR in both female and male mice. No major differences were identified between the female and male mice, except a few variable magnitudes in *Gdf10*, *Fgf21*, and *Hamp2*. Although we reported the functional analysis of the cerulein-induced CP and recovery mouse model previously [10], further investigation is warranted for the identified DEGs in CP and CP recovery for mechanistic exploration and therapeutic development.

## 5. Conclusion

Our RNA-seq profile analysis revealed more than 3,600 DEGs in the cerulein-induced CP and the recovery animal model, demonstrating a characteristic transcriptional modification or signatures for the disease recovery, with 3,600 DEGs in CP vs. 166 in CPR. From a biological standpoint, these data offer an opportunity to better understand the transcriptomic mechanisms through which CP develops and then recovers. Further investigation is warranted for exploring the functional contribution of the identified DEGs critical for the disease recovery and repair, which may lead to identification of molecular targets for therapeutic development against CP.

## Figures and Tables

**Table 1:** DEGs shared between CP and CPR.

Group^∗^	No. of DEGs	DEGs^∗∗^
UP in CP and CPR	96	*Cilp*, *Pgc*, *Fetub*, *Kcnj15*, *Sfrp1*, *Myoc*, *Obp2b*, *Gm830*, *Ptn*, *Gdf10*, *Pdgfrl*, *Hmcn2*, *Lbp*, *Cpxm2*, *Inmt*, *Plod2*, *Hmcn1*, *Ephb2*, *Cygb*, *Fam107b*, *Nkd2*, *Slit2*, *Itgbl1*, *Epha3*, *Olfml3*, *Igsf9*, *Zfp521*, *Ms4a6d*, *Prune2*, *Sparcl1*, *C7*, *Gypc*, *Ngfr*, *Casp1*, *Tmem45a*, *Wnt5a*, *Klhl29*, *Mfap4*, *Reg3a*, *Mgp*, *Stum*, *Reg3g*, *Zim1*, *Ccr2*, *Pcsk5*, *Gm10334*, *Vgll3*, *Gfpt2*, *Galnt15*, *Ifi211*, *Prox1*, *Plekho2*, *Mdk*, *Slc39a10*, *Dst*, *Eln*, *Nfam1*, *Pld1*, *Fbln1*, *Mrc1*, *Flt3l*, *Prss1*, *Fam102b*, *Ifi207*, *Cacna1c*, *Sult5a1*, *C1qtnf7*, *Ear2*, *Ogn*, *Reln*, *Nox4*, *Gli1*, *Lyl1*, *Retnla*, *Gask1b*, *Zfhx4*, *Ahnak*, *Elmo1*, *Cdkn1c*, *Bgn*, *Sulf2*, *Pla2g7*, *Renbp*, *Fes*, *Numbl*, *Ms4a7*, *Pcdh18*, *Kcnb1*, *Prrg3*, *Tenm4*, *Lgals9*, *Stab1*, *Ckb*, *Col14a1*, *Olfml2b*, *Fcrls*

DN in CP and CPR	24	*1810007D17Rik*, *Nxph2*, *Amy2-ps1*, *Smtnl2*, *Iyd*, *Cldn9*, *Dclk3*, *Gal*, *Cela3a*, *Cdhr2*, *Scn4b*, *Camk2b*, *Cyp3a13*, *Tulp2*, *Itih4*, *Smim24*, *Fgf21*, *Nat8f1*, *Padi2*, *Slc38a5*, *Hamp2*, *Sypl2*, *Vtn*, *Ctrl*

DN in CP, UP in CPR	1	*Reg2*

UP in CP, DN in CPR	11	*Fxyd1*, *Upp2*, *Slc6a4*, *Abhd11os*, *Susd2*, *Dpf1*, *Usp2*, *Sdr9c7*, *Pkd1l2*, *Apip*, *Try10*

Total	132	

^∗^UP: upregulated; DN: downregulated. ^∗∗^DEGs were listed in an order of Log2(fold change) value high to low.

**Table 2:** DEGs specific to CPR.

Group^∗^	No. of DEGs	DEGs^∗∗^
UP in CPR	31	*Agr2*, *Thbs4*, *Lyz1*, *D630003M21Rik*, *Plekhh2*, *Slc6a2*, *Prr33*, *Cldn2*, *Tmod2*, *Tnfrsf18*, *Col8a2*, *Hap1*, *Mboat1*, *Eif5a2*, *Dnm1*, *3110039I08Rik*, *Sema6a*, *Golm1*, *Coro2a*, *Rnasel*, *Wdr78*, *Ptgir*, *Pde4b*, *Speg*, *Dnm3*, *Elovl6*, *Nrp2*, *Slc37a2*, *Pde3a*, *Piezo2*, *Dbp*

DN in CPR	3	*Nsg1*, *Car4*, *Clec11a*

Total	34	

^∗^UP: upregulated; DN: downregulated. ^∗∗^DEGs were listed in an order of Log2(fold change) value high to low.

**Table 3:** Top 20 gene sets enriched in 115 DEGs shared between CP and CPR^∗^.

Gene set	No. of DEGs (%)	-Log10(FDR)	DEGs	Ref.
NABA MATRISOME	30 (26.1)	22.806	*Bgn*, *C1qtnf7*, *Cela3a*, *Cilp*, *Col14a1*, *Fbln1*, *Fgf21*, *Flt3lg*, *Gdf10*, *Hmcn1*, *Hmcn2*, *Itih4*, *Lgals9b*, *Mdk*, *Mfap4*, *Mgp*, *Ogn*, *Pcsk5*, *Plod2*, *Prss1*, *Ptn*, *Reg1b*, *Reg3g*, *Reln*, *Sfrp1*, *Slit2*, *Sparcl1*, *Sulf2*, *Vtn*, *Wnt5a*	[35]

BOQUEST STEM CELL UP	18 (15.7)	19.127	*C7*, *Cilp*, *Col14a1*, *Fbln1*, *Fxyd1*, *Gdf10*, *Gfpt2*, *Itgbl1*, *Mfap4*, *Mgp*, *Myoc*, *Ogn*, *Olfml2b*, *Olfml3*, *Pdgfrl*, *Slit2*, *Tmem45a*, *Wnt5a*	[43]

CHEN_METABOLIC_SYNDROM_NETWORK	24 (20.9)	14.042	*Bgn*, *Casp1*, *Col14a1*, *Cygb*, *Fes*, *Fetub*, *Gask1b*, *Gdf10*, *Ifi16*, *Itgbl1*, *Lyl1*, *Mrc1*, *Ms4a6a*, *Ms4a7*, *Nfam1*, *Numbl*, *Olfml3*, *Padi2*, *Pdgfrl*, *Pla2g7*, *Renbp*, *Stab1*, *Vtn*, *Znf521*	[44]

CHICAS_RB1_TARGETS_CONFLUENT	18 (15.7)	13.430	*Ahnak*, *Bgn*, *C7*, *Ephb2*, *Fbln1*, *Galnt15*, *Gask1b*, *Ifi16*, *Itgbl1*, *Mdk*, *Mfap4*, *Olfml3*, *Pdgfrl*, *Ptn*, *Reln*, *Sfrp1*, *Tenm4*, *Vgll3*	[36]

SMID_BREAST_CANCER_NORMAL_LIKE_UP	17 (14.8)	13.430	*C7*, *Casp1*, *Ccr2*, *Col14a1*, *Elmo1*, *Flt3lg*, *Fxyd1*, *Ifi16*, *Lyl1*, *Mfap4*, *Mrc1*, *Ms4a6a*, *Pcsk5*, *Ptn*, *Reln*, *Sfrp1*, *Sparcl1*	[37]

NABA_CORE_MATRISOME	13 (11.3)	11.257	*Bgn*, *Cilp*, *Col14a1*, *Fbln1*, *Hmcn1*, *Hmcn2*, *Mfap4*, *Mgp*, *Ogn*, *Reln*, *Slit2*, *Sparcl1*, *Vtn*	[35]

DELYS_THYROID_CANCER_DN	12 (10.4)	10.732	*Elmo1*, *Epha3*, *Fbln1*, *Fxyd1*, *Gdf10*, *Mgp*, *Myoc*, *Ogn*, *Pla2g7*, *Ptn*, *Reln*, *Sfrp1*	[38]

NABA_MATRISOME_ASSOCIATED	17 (14.8)	10.353	*C1qtnf7*, *Cela3a*, *Fgf21*, *Flt3lg*, *Gdf10*, *Itih4*, *Lgals9b*, *Mdk*, *Pcsk5*, *Plod2*, *Prss1*, *Ptn*, *Reg1b*, *Reg3g*, *Sfrp1*, *Sulf2*, *Wnt5a*	[35]

SCHAEFFER_PROSTATE_DEVELOPMENT_48HR_DN	14 (12.2)	10.300	*C1qtnf7*, *Cpxm2*, *Ephb2*, *Fam107b*, *Fbln1*, *Gypc*, *Lbp*, *Mfap4*, *Ms4a7*, *Pcdh18*, *Prox1*, *Slit2*, *Sparcl1*, *Znf521*	[39]

LIM_MAMMARY_STEM_CELL_UP	14 (12.2)	9.564	*Col14a1*, *Cygb*, *Fbln1*, *Fxyd1*, *Gypc*, *Klhl29*, *Ngfr*, *Pcdh18*, *Pla2g7*, *Reln*, *Scn4b*, *Slc38a5*, *Slit2*, *Vgll3*	[45]

ONDER_CDH1_TARGETS_2_UP	11 (9.6)	8.959	*Bgn*, *Ephb2*, *Fbln1*, *Gypc*, *Itgbl1*, *Olfml3*, *Pdgfrl*, *Plod2*, *Prune2*, *Wnt5a*, *Zfhx4*	[46]

SCHUETZ_BREAST_CANCER_DUCTAL_INVASIVE_UP	12 (10.4)	8.846	*Bgn*, *Casp1*, *Cilp*, *Col14a1*, *Gfpt2*, *Ifi16*, *Itgbl1*, *Ms4a6a*, *Nox4*, *Olfml2b*, *Olfml3*, *Sparcl1*	[40]

NABA_ECM_GLYCOPROTEINS	10 (8.7)	8.758	*Cilp*, *Fbln1*, *Hmcn1*, *Hmcn2*, *Mfap4*, *Mgp*, *Reln*, *Slit2*, *Sparcl1*, *Vtn*	[35]

LEE_BMP2_TARGETS_UP	15 (13.0)	8.396	*Casp1*, *Cilp*, *Ckb*, *Cpxm2*, *Fam102b*, *Fxyd1*, *Galnt15*, *Mfap4*, *Mgp*, *Ngfr*, *Nkd2*, *Pld1*, *Renbp*, *Vtn*, *Znf521*	[47]

WEST_ADRENOCORTICAL_TUMOR_DN	13 (11.3)	7.912	*Ahnak*, *Bgn*, *C7*, *Fbln1*, *Fxyd1*, *Mgp*, *Ngfr*, *Ogn*, *Olfml3*, *Pld1*, *Plekho2*, *Stab1*, *Vtn*	[41]

VECCHI_GASTRIC_CANCER_ADVANCED_VS_EARLY_UP	9 (7.8)	7.818	*Bgn*, *Gfpt2*, *Itgbl1*, *Mgp*, *Nox4*, *Pla2g7*, *Plod2*, *Slit2*, *Tmem45a*	[42]

SMID_BREAST_CANCER_LUMINAL_B_DN	13 (11.3)	7.802	*C7*, *Ccr2*, *Col14a1*, *Fbln1*, *Gal*, *Ifi16*, *Lbp*, *Mfap4*, *Mrc1*, *Padi2*, *Ptn*, *Sfrp1*, *Tmem45a*	[37]

SMID_BREAST_CANCER_BASAL_DN	14 (12.2)	7.788	*Ahnak*, *Camk2b*, *Cilp*, *Col14a1*, *Epha3*, *Fbln1*, *Gask1b*, *Itgbl1*, *Mfap4*, *Ogn*, *Olfml3*, *Pcsk5*, *Slc6a4*, *Sparcl1*	[37]

FLORIO_NEOCORTEX_BASAL_RADIAL_GLIA_UP	11 (9.6)	7.362	*C1qtnf7*, *C7*, *Ccr2*, *Cela3a*, *Epha3*, *Itgbl1*, *Iyd*, *Kcnj15*, *Pcdh18*, *Slc6a4*, *Wnt5a*	[48]

KIM_GLIS2_TARGETS_UP	7 (6.1)	7.219	*Ccr2*, *Col14a1*, *Mfap4*, *Mgp*, *Ogn*, *Pdgfrl*, *Sparcl1*	[49]

^∗^Only 115 out of the 132 DEGs were found in those gene sets for the enrichment analysis.

**Table 4:** Top 10 gene sets derived from the 32 unique DEGs in CPR^∗^.

Gene set	No. of DEGs (%)	-Log10(FDR)	DEGs	Ref.
DURCHDEWALD_SKIN_CARCINOGENESIS_UP	4 (12.5)	3.194	*Dbp*, *Elovl6*, *Plekhh2*, *Tnfrsf18*	[52]
YOSIMURA_MAPK8_TARGETS_UP	8 (25.0)	3.194	*Clec11a*, *Dbp*, *Hap1*, *Nsg1*, *Pde3a*, *Pde4b*, *Slc6a2*, *Thbs4*	[53]
FEVR_CTNNB1_TARGETS_UP	6 (18.8)	2.689	*Agr2*, *Ca4*, *Coro2a*, *Dbp*, *Elovl6*, *Nrp2*	[54]
FIGUEROA_AML_METHYLATION_CLUSTER_2_UP	3 (9.4)	2.431	*Dnm3*, *Nrp2*, *Thbs4*	[50]
MEISSNER_NPC_HCP_WITH_H3_UNMETHYLATED	5 (15.6)	2.259	*Ca4*, *Col8a2*, *Slc6a2*, *Speg*, *Thbs4*	[51]
ONKEN_UVEAL_MELANOMA_DN	5 (15.6)	2.259	*Dbp*, *Nrp2*, *Nsg1*, *Pde4b*, *Sema6a*	[55]
PLASARI TGFB1 TARGETS 10HR DN	4 (12.5)	2.259	*Dbp*, *Elovl6*, *Plekhh2*, *Rnasel*	[56]
LEIN_MIDBRAIN_MARKERS	3 (9.4)	2.184	*Dnm3*, *Eif5a2*, *Hap1*	[57]
CHANDRAN_METASTASIS_DN	4 (12.5)	2.041	*Col8a2*, *Golm1*, *Pde4b*, *Thbs4*	[58]
GOBERT_OLIGODENDROCYTE_DIFFERENTIATION_DN	6 (18.8)	2.041	*Dbp*, *Dnm3*, *Mboat1*, *Pde4b*, *Rnasel*, *Speg*	[59]

^∗^Only 32 out of the 34 DEGs were found in those gene sets for the enrichment analysis.

## Data Availability

The RNA-seq data used to support the findings of this study are included within the supplementary information file Table S1 and can be accessed at https://www.uth.edu/bioinfo/Cao_RNA-seq.xlsx data.

## Supplementary Materials

Supplementary 1 Supplementary Table S1: a complete list of all identified DEGs.

Supplementary 2 Supplementary Table S2: mRNA level by qPCR assay presented as [2^−(ΔCt)^] × 10^5^.

## References

[1] Go V., Everhart J. (1994). *Pancreatitis*.

[2] Yadav D., Hawes R. H., Brand R. E. (2009). Alcohol consumption, cigarette smoking, and the risk of recurrent acute and chronic pancreatitis. *Archives of Internal Medicine*.

[3] Irving H. M., Samokhvalov A. V., Rehm J. (2009). Alcohol as a risk factor for pancreatitis. A systematic review and meta-analysis. *Journal of the Pancreas*.

[4] Yadav D., Timmons L., Benson J. T., Dierkhising R. A., Chari S. T. (2011). Incidence, prevalence, and survival of chronic pancreatitis: a population-based study. *The American Journal of Gastroenterology*.

[5] Majumder S., Chari S. T. (2016). Chronic pancreatitis. *The Lancet*.

[6] Peery A. F., Crockett S. D., Barritt A. S. (2015). Burden of gastrointestinal, liver, and pancreatic diseases in the United States. *Gastroenterology*.

[7] Hart P. A., Bellin M. D., Andersen D. K. (2016). Type 3c (pancreatogenic) diabetes mellitus secondary to chronic pancreatitis and pancreatic cancer. *The lancet Gastroenterology & hepatology*.

[8] Lowenfels A. B., Maisonneuve P., Cavallini G. (1993). Pancreatitis and the risk of pancreatic cancer. *The New England Journal of Medicine*.

[9] Comfort M., Gambill E., Baggenstoss A. (1946). Chronic relapsing pancreatitis; a study of 29 cases without associated disease of the biliary or gastrointestinal tract. *Gastroenterology*.

[10] Obafemi T. F., Yu P., Li J. (2018). Comparable responses in male and female mice to cerulein-induced chronic pancreatic injury and recovery. *Journal of the Pancreas*.

[11] Wang Z., Gerstein M., Snyder M. (2009). RNA-Seq: a revolutionary tool for transcriptomics. *Nature Reviews Genetics*.

[12] Hua X., Wang Y. Y., Jia P. (2020). Multi-level transcriptome sequencing identifies COL1A1 as a candidate marker in human heart failure progression. *BMC Medicine*.

[13] Hrdlickova R., Toloue M., Tian B. (2017). RNA-Seq methods for transcriptome analysis. *Wiley Interdisciplinary Reviews*.

[14] Nagalakshmi U., Waern K., Snyder M. (2010). RNA-Seq: a method for comprehensive transcriptome analysis. *Current Protocols in Molecular Biology*.

[15] Yang L., Zhao H., Yin X. (2020). Exploring cisplatin resistance in ovarian cancer through integrated bioinformatics approach and overcoming chemoresistance with sanguinarine. *American Journal of Translational Research*.

[16] Chen X., Chang J. T. (2017). Planning bioinformatics workflows using an expert system. *Bioinformatics*.

[17] Bolger A. M., Lohse M., Usadel B. (2014). Trimmomatic: a flexible trimmer for Illumina sequence data. *Bioinformatics*.

[18] Dobin A., Davis C. A., Schlesinger F. (2013). STAR: ultrafast universal RNA-seq aligner. *Bioinformatics*.

[19] Li B., Ruotti V., Stewart R. M., Thomson J. A., Dewey C. N. (2010). RNA-Seq gene expression estimation with read mapping uncertainty. *Bioinformatics*.

[20] Anders S., Pyl P. T., Huber W. (2015). HTSeq--a Python framework to work with high-throughput sequencing data. *Bioinformatics*.

[21] DePristo M. A., Banks E., Poplin R. (2011). A framework for variation discovery and genotyping using next-generation DNA sequencing data. *Nature Genetics*.

[22] Love M. I., Huber W., Anders S. (2014). Moderated estimation of fold change and dispersion for RNA-seq data with DESeq2. *Genome Biology*.

[23] Chang J. T., Nevins J. R. (2006). GATHER: a systems approach to interpreting genomic signatures. *Bioinformatics*.

[24] Liberzon A., Subramanian A., Pinchback R., Thorvaldsdottir H., Tamayo P., Mesirov J. P. (2011). Molecular signatures database (MSigDB) 3.0. *Bioinformatics*.

[25] Cao Y., Chen L., Zhang W. (2007). Identification of apoptotic genes mediating TGF-beta/Smad3-induced cell death in intestinal epithelial cells using a genomic approach. *American Journal of Physiology Gastrointestinal and Liver Physiology*.

[26] Bou-Gharios G., Garrett L. A., Rossert J. (1996). A potent far-upstream enhancer in the mouse pro alpha 2(I) collagen gene regulates expression of reporter genes in transgenic mice. *The Journal of Cell Biology*.

[27] Jaster R. (2004). Molecular regulation of pancreatic stellate cell function. *Molecular Cancer*.

[28] Denton C. P., Zheng B., Evans L. A. (2003). Fibroblast-specific expression of a kinase-deficient type II transforming growth factor beta (TGFbeta) receptor leads to paradoxical activation of TGFbeta signaling pathways with fibrosis in transgenic mice. *The Journal of biological chemistry*.

[29] Zebedee Z., Hara E. (2001). Id proteins in cell cycle control and cellular senescence. *Oncogene*.

[30] Cunningham N. S., Jenkins N. A., Gilbert D. J., Copeland N. G., Reddi A. H., Lee S. J. (1995). Growth/differentiation factor-10: a new member of the transforming growth factor-beta superfamily related to bone morphogenetic protein-3. *Growth Factors*.

[31] Kim S., Choe S., Lee D. K. (2016). BMP-9 enhances fibroblast growth factor 21 expression and suppresses obesity. *Biochimica et Biophysica Acta*.

[32] Lu S., Seravalli J., Harrison-Findik D. (2015). Inductively coupled mass spectrometry analysis of biometals in conditional Hamp1 and Hamp1 and Hamp2 transgenic mouse models. *Transgenic Research*.

[33] Li S., Li R., Wang H., Li L., Li H., Li Y. (2018). The key genes of chronic pancreatitis which bridge chronic pancreatitis and pancreatic cancer can be therapeutic targets. *Pathology Oncology Research*.

[34] Tu J., Huang Z., Wang Y. (2021). Transcriptome analysis of the procession from chronic pancreatitis to pancreatic cancer and metastatic pancreatic cancer. *Scientific Reports*.

[35] Naba A., Clauser K. R., Hoersch S., Liu H., Carr S. A., Hynes R. O. (2012). Proteomics of Normal and Tumor Extracellular Matrices. *Molecular & Cellular Proteomics*.

[36] Chicas A., Wang X., Zhang C. (2010). Dissecting the unique role of the retinoblastoma tumor suppressor during cellular senescence. *Cancer Cell*.

[37] Smid M., Wang Y., Zhang Y. (2008). Subtypes of breast cancer show preferential site of relapse. *Cancer Research*.

[38] Delys L., Detours V., Franc B. (2007). Gene expression and the biological phenotype of papillary thyroid carcinomas. *Oncogene*.

[39] Schaeffer E. M., Marchionni L., Huang Z. (2008). Androgen-induced programs for prostate epithelial growth and invasion arise in embryogenesis and are reactivated in cancer. *Oncogene*.

[40] Schuetz C. S., Bonin M., Clare S. E. (2006). Progression-specific genes identified by expression profiling of matched ductal carcinomas in situ and invasive breast tumors, combining laser capture microdissection and oligonucleotide microarray analysis. *Cancer Research*.

[41] West A. N., Neale G. A., Pounds S. (2007). Gene expression profiling of childhood adrenocortical tumors. *Cancer Research*.

[42] Vecchi M., Nuciforo P., Romagnoli S. (2007). Gene expression analysis of early and advanced gastric cancers. *Oncogene*.

[43] Boquest A. C., Shahdadfar A., Fronsdal K. (2005). Isolation and transcription profiling of purified uncultured human stromal stem cells: alteration of gene expression after in vitro cell culture. *Molecular Biology of the Cell*.

[44] Chen Y., Zhu J., Lum P. Y. (2008). Variations in DNA elucidate molecular networks that cause disease. *Nature*.

[45] Lim E., Wu D., Pal B. (2010). Transcriptome analyses of mouse and human mammary cell subpopulations reveal multiple conserved genes and pathways. *Breast Cancer Research*.

[46] onder T. T., Gupta P. B., Mani S. A., Yang J., Lander E. S., Weinberg R. A. (2008). Loss of E-cadherin promotes metastasis via multiple downstream transcriptional pathways. *Cancer Research*.

[47] Lee K. Y., Jeong J. W., Wang J. (2007). Bmp2 is critical for the murine uterine decidual response. *Molecular and Cellular Biology*.

[48] Florio M., Albert M., Taverna E. (2015). Human-specific gene ARHGAP11B promotes basal progenitor amplification and neocortex expansion. *Science*.

[49] Kim Y. S., Kang H. S., Herbert R. (2008). Kruppel-like zinc finger protein Glis2 is essential for the maintenance of normal renal functions. *Molecular and Cellular Biology*.

[50] Figueroa M. E., Lugthart S., Li Y. (2010). DNA methylation signatures identify biologically distinct subtypes in acute myeloid leukemia. *Cancer Cell*.

[51] Meissner A., Mikkelsen T. S., Gu H. (2008). Genome-scale DNA methylation maps of pluripotent and differentiated cells. *Nature*.

[52] Durchdewald M., Guinea-Viniegra J., Haag D. (2008). Podoplanin is a novel fos target gene in skin carcinogenesis. *Cancer Research*.

[53] Yoshimura K., Aoki H., Ikeda Y. (2005). Regression of abdominal aortic aneurysm by inhibition of c-Jun N-terminal kinase. *Nature Medicine*.

[54] Fevr T., Robine S., Louvard D., Huelsken J. (2007). Wnt/beta-catenin is essential for intestinal homeostasis and maintenance of intestinal stem cells. *Molecular and Cellular Biology*.

[55] Onken M. D., Ehlers J. P., Worley L. A., Makita J., Yokota Y., Harbour J. W. (2006). Functional gene expression analysis uncovers phenotypic switch in aggressive uveal melanomas. *Cancer Research*.

[56] Plasari G., Calabrese A., Dusserre Y. (2009). Nuclear factor I-C links platelet-derived growth factor and transforming growth factor beta1 signaling to skin wound healing progression. *Molecular and Cellular Biology*.

[57] Lein E. S., Hawrylycz M. J., Ao N. (2007). Genome-wide atlas of gene expression in the adult mouse brain. *Nature*.

[58] Chandran U. R., Ma C., Dhir R. (2007). Gene expression profiles of prostate cancer reveal involvement of multiple molecular pathways in the metastatic process. *BMC Cancer*.

[59] Pescini Gobert R., Joubert L., Curchod M. L. (2009). Convergent functional genomics of oligodendrocyte differentiation identifies multiple autoinhibitory signaling circuits. *Molecular and Cellular Biology*.

[60] Vonlaufen A., Phillips P. A., Xu Z. (2011). Withdrawal of alcohol promotes regression while continued alcohol intake promotes persistence of LPS-induced pancreatic injury in alcohol-fed rats. *Gut*.

[61] Witjas F. M. R., van den Berg B. M., van den Berg C. W., Engelse M. A., Rabelink T. J. (2019). Concise review: the endothelial cell extracellular matrix regulates tissue homeostasis and repair. *Stem Cells Translational Medicine*.

[62] McKee T. J., Perlman G., Morris M., Komarova S. V. (2019). Extracellular matrix composition of connective tissues: a systematic review and meta-analysis. *Scientific Reports*.

[63] Minton K. (2014). Extracellular matrix: preconditioning the ECM for fibrosis. *Nature Reviews Molecular Cell Biology*.

[64] Nguyen-Ngoc K. V., Cheung K. J., Brenot A. (2012). ECM microenvironment regulates collective migration and local dissemination in normal and malignant mammary epithelium. *Proceedings of the National Academy of Sciences of the United States of America*.

[65] Zschenker O., Streichert T., Hehlgans S., Cordes N. (2012). Genome-wide gene expression analysis in cancer cells reveals 3D growth to affect ECM and processes associated with cell adhesion but not DNA repair. *PLoS One*.

[66] Zhang C. L., Zhao Q., Liang H. (2018). Cartilage intermediate layer protein-1 alleviates pressure overload-induced cardiac fibrosis via interfering TGF-beta1 signaling. *Journal of Molecular and Cellular Cardiology*.

[67] Zhou T., Yu L., Huang J. (2019). GDF10 inhibits proliferation and epithelial-mesenchymal transition in triple-negative breast cancer via upregulation of Smad7. *Aging*.

[68] Luo Y., Yang Y., Liu M. (2019). Oncogenic KRAS reduces expression of FGF21 in acinar cells to promote pancreatic tumorigenesis in mice on a high-fat diet. *Gastroenterology*.

[69] Raghuraman S., Donkin I., Versteyhe S., Barrès R., Simar D. (2016). The emerging role of epigenetics in inflammation and immunometabolism. *Trends in Endocrinology and Metabolism*.

[70] Tan A. C., Jimeno A., Lin S. H. (2009). Characterizing DNA methylation patterns in pancreatic cancer genome. *Molecular Oncology*.

[71] Natale F., Vivo M., Falco G., Angrisano T. (2019). Deciphering DNA methylation signatures of pancreatic cancer and pancreatitis. *Clinical Epigenetics*.

[72] Shindo K., Aishima S., Ohuchida K. (2013). Podoplanin expression in cancer-associated fibroblasts enhances tumor progression of invasive ductal carcinoma of the pancreas. *Molecular Cancer*.

[73] Kitano H., Kageyama S., Hewitt S. M. (2010). Podoplanin expression in cancerous stroma induces lymphangiogenesis and predicts lymphatic spread and patient survival. *Archives of Pathology & Laboratory Medicine*.

[74] Weston C. R., Davis R. J. (2007). The JNK signal transduction pathway. *Current Opinion in Cell Biology*.

[75] Minutoli L., Altavilla D., Marini H. (2004). Protective effects of SP600125 a new inhibitor of c-Jun N-terminal kinase (JNK) and extracellular-regulated kinase (ERK1/2) in an experimental model of cerulein-induced pancreatitis. *Life Sciences*.

[76] Kim D. G., Bae G. S., Choi S. B. (2015). Guggulsterone attenuates cerulein-induced acute pancreatitis via inhibition of ERK and JNK activation. *International Immunopharmacology*.

[77] Choi S. B., Bae G. S., Jo I. J., Wang S., Song H. J., Park S. J. (2016). Berberine inhibits inflammatory mediators and attenuates acute pancreatitis through deactivation of JNK signaling pathways. *Molecular Immunology*.

[78] An W., Zhu J. W., Jiang F. (2020). Fibromodulin is upregulated by oxidative stress through the MAPK/AP-1 pathway to promote pancreatic stellate cell activation. *Pancreatology*.

[79] Murtaugh L. C. (2008). The what, where, when and how of Wnt/*β*-catenin signaling in pancreas development. *Organogenesis*.

[80] Huang H. L., Tang G. D., Liang Z. H. (2019). Role of Wnt/*β*-catenin pathway agonist SKL2001 in caerulein-induced acute pancreatitis. *Canadian Journal of Physiology and Pharmacology*.

[81] Cao Y., Liu X., Zhang W. (2009). TGF-beta repression of Id2 induces apoptosis in gut epithelial cells. *Oncogene*.

[82] Vogelmann R., Ruf D., Wagner M., Adler G., Menke A. (2001). Effects of fibrogenic mediators on the development of pancreatic fibrosis in a TGF-beta1 transgenic mouse model. *American Journal of Physiology Gastrointestinal and Liver Physiology*.

[83] Sato T., Shibata W., Maeda S. (2019). Adhesion molecules and pancreatitis. *Journal of Gastroenterology*.

[84] Greco S. A., Chia J., Inglis K. J. (2010). Thrombospondin-4 is a putative tumour-suppressor gene in colorectal cancer that exhibits age-related methylation. *BMC Cancer*.

[85] Brychtova V., Vojtesek B., Hrstka R. (2011). Anterior gradient 2: a novel player in tumor cell biology. *Cancer Letters*.

[86] Ramachandran V., Arumugam T., Wang H., Logsdon C. D. (2008). Anterior gradient 2 is expressed and secreted during the development of pancreatic cancer and promotes cancer cell survival. *Cancer Research*.

[87] Huszarik K., Wright B., Keller C. (2010). Adjuvant immunotherapy increases beta cell regenerative factor Reg2 in the pancreas of diabetic mice. *Journal of Immunology*.

[88] Li Q., Li B., Miao X., Ramgattie C., Gao Z. H., Liu J. L. (2017). Reg2 expression is required for pancreatic islet compensation in response to aging and high-fat diet-induced obesity. *Endocrinology*.

[89] Lunova M., Schwarz P., Nuraldeen R. (2017). Hepcidin knockout mice spontaneously develop chronic pancreatitis owing to cytoplasmic iron overload in acinar cells. *The Journal of Pathology*.

[90] Gao X., Cao Y., Staloch D. A. (2014). Bone morphogenetic protein signaling protects against cerulein-induced pancreatic fibrosis. *PLoS One*.

[91] Gao X., Cao Y., Yang W. (2013). BMP2 inhibits TGF-beta-induced pancreatic stellate cell activation and extracellular matrix formation. *American Journal of Physiology Gastrointestinal and Liver Physiology*.

[92] Lunova M., Goehring C., Kuscuoglu D. (2014). Hepcidin knockout mice fed with iron-rich diet develop chronic liver injury and liver fibrosis due to lysosomal iron overload. *Journal of Hepatology*.

[93] Boggs K., Wang T., Orabi A. I. (2018). Pancreatic gene expression during recovery after pancreatitis reveals unique transcriptome profiles. *Scientific Reports*.

[94] Zucker I., Beery A. K. (2010). Males still dominate animal studies. *Nature*.

